# Relationship between postinterventional cerebral hyperdensities and malignant brain edema in patients with acute ischemic stroke after mechanical thrombectomy

**DOI:** 10.3389/fneur.2025.1693606

**Published:** 2025-10-03

**Authors:** Xiaocui Wang, Junhao Du, Yage Zhao, Zhiliang Guo, Jie Hou, Huaishun Wang, Guangyi Zhou, Guodong Xiao

**Affiliations:** ^1^Department of Neurology, The Second Affiliated Hospital of Soochow University, Suzhou, China; ^2^Department of Neruology, Jinan City People’s Hospital, Jinan, China; ^3^Department of Neurology, The Affiliated Nanhua Hospital, Hengyang Medical School, University of South China, Hengyang, China

**Keywords:** acute ischemic stroke, mechanical thrombectomy, postinterventional cerebral hyperdensities, malignant brain edema, nomogram

## Abstract

**Background:**

This study aimed to evaluate the predictive value of postinterventional cerebral hyperdensities (PCHDs) for malignant brain edema (MBE) in acute ischemic stroke (AIS) patients after mechanical thrombectomy (MT). We sought to establish and validate a nomogram for predicting MBE in this population.

**Methods:**

This study included patients with acute anterior circulation large vessel occlusion stroke treated with MT at our hospital from May 2017 to July 2024. PCHDs were classified based on their distribution characteristics and extent. A multivariate logistic regression analysis was used to assess the predictive value of PCHDs subtypes for MBE. Receiver operating characteristic (ROC) curve analysis identified optimal predictive thresholds. Least absolute shrinkage and selection operator regression was applied to select significant predictors of MBE to construct a nomogram. The nomogram’s performance was evaluated using the C-index, calibration plots, and decision curve analysis.

**Results:**

Among 516 enrolled patients, 126 (24.4%) developed MBE. The cortex sign [adjusted odds ratio (OR) = 6.290, 95% confidence interval (CI): 2.581–15.329], basal ganglia sign (adjusted OR = 4.081, 95% CI: 1.831–9.096), and combined sign (adjusted OR = 8.295, 95% CI: 3.942–17.454) were independent risk factors for MBE. Higher PCHDs scores were inversely associated with MBE risk (adjusted OR = 0.620, 95% CI: 0.534–0.719). The nomogram incorporating age, atrial fibrillation, baseline National Institutes of Health Stroke Scale (NIHSS) score, occlusion site, white blood cell (WBC) count, total cholesterol level, and PCHDs score demonstrated good discrimination (C-index: 0.904) and calibration (Hosmer–Lemeshow test, *p* = 0.851) to predict MBE.

**Conclusion:**

PCHDs show a strong association with MBE in AIS patients. Our nomogram provides individualized prediction of post-MT MBE risk; however, multicenter validation is warranted.

## Introduction

Acute ischemic stroke (AIS) is characterized by high morbidity, mortality, and disability rates. The primary treatment goal for AIS is rapid recanalization of occluded vessels to salvage the ischemic penumbra. Since 2015, significant advancements in endovascular treatment have been achieved (1–5), offering advantages such as rapid recanalization, reduced hemorrhagic transformation rates, and extended therapeutic time windows.

Although endovascular treatment rapidly restores blood perfusion, reduces malignant brain edema (MBE) incidence, and improves prognosis in AIS patients, MBE still occurs in 10–78% of cases, peaking at 2–5 days post-ischemia, with mortality rates approaching 80% (6–8). MBE is characterized by rapid neurological deterioration due to massive cerebral edema, resulting in transtentorial herniation, death, or poor functional outcomes (9). However, there are limited treatment options for MBE. Previous randomized controlled trials showed that early decompressive craniectomy can reduce mortality and increase the chance of a good functional outcome (10). Therefore, early prediction of MBE after recanalization is critical to enhance perioperative patient management.

In clinical practice, postprocedural brain computed tomography (CT) is routinely performed to assess MBE risk and guide management. Postinterventional cerebral hyperdensities (PCHDs) are common imaging findings on post-thrombectomy CT scans and may result from hemorrhage and/or contrast extravasation. Multiple studies (11–13) have demonstrated significant associations between PCHDs and hemorrhagic transformation or neurological deterioration, suggesting their utility as poor prognostic indicators. However, Parrilla et al. (14) found no statistically significant association between PCHDs and clinical outcomes. The possible reasons for such a wide variation in the results may be attributed to discrepancies in the definition of hyperdense lesions, as well as differences in evaluation methods, grouping criteria, or classification methods.

The Alberta Stroke Program Early CT Score (ASPECTS) (15), a semiquantitative tool dividing the middle cerebral artery (MCA) territory into 10 regions, effectively evaluates early ischemic changes. Previous studies applied ASPECTS to assess the scope of PCHDs distribution for the semi-quantitative analysis, which was simple, convenient, rapid, objective, and more accurate. While several studies have evaluated the correlation between PCHDs and hemorrhagic transformation or final cerebral infarction volume, fewer have explored their relationship with MBE. This study aimed to explore the relationship between PCHDs and MBE using different classification methods of PCHDs. By assessing the predictive value of PCHDs for MBE, we sought to develop and validate a nomogram for predicting MBE after mechanical thrombectomy (MT) in AIS patients.

## Methods

### Study design and participants

This retrospective study was approved by the Institutional Review Board of the Second Affiliated Hospital of Soochow University (approval number: JD-LK-2022-140-01). The requirement for informed consent was waived.

This study included patients with acute anterior circulation large vessel occlusion stroke who underwent MT in the Department of Neurology of the Second Affiliated Hospital of Soochow University between May 2017 and July 2024. The inclusion criteria were as follows: (1) age ≥ 18 years; (2) no hemorrhage confirmed by preoperative brain CT; and (3) internal carotid artery (ICA) or proximal MCA occlusion (M1/M2 segment) treated with MT. The exclusion criteria were as follows: (1) patients who did not undergo brain CT examination immediately (within 1 h) after MT; (2) patients with other life-threatening comorbidities, such as malignant tumors or severe organ failure; and (3) patients with missing clinical data, imaging data, or 3-month outcome follow-up data.

### Data collection and definitions

Demographics, laboratory data, procedural characteristics, and imaging findings were collected for analysis. Demographics were collected from medical records. Laboratory data, including routine blood tests and biochemical tests, were obtained from peripheral venous blood samples collected after admission. Imaging data, including ASPECTS, PCHDs, and MBE, were extracted from preoperative and postoperative brain CT scans.

Stroke etiology was classified according to the Trial of Org 10,712 in Acute Stroke Treatment (TOAST) etiology (16). Stroke severity was assessed at admission with the National Institutes of Health Stroke Scale (NIHSS) (17). ASPECTS was utilized to assess early cerebral ischemic changes on preoperative brain CT (15). Successful reperfusion was defined as modified thrombolysis in cerebral infarction (mTICI) of 2b-3 grade on digital subtraction angiography (DSA) (18). According to the modified European Cooperative Acute Stroke Study II (ECASS-II) criteria (19), symptomatic intracranial hemorrhage (sICH) was defined as blood at any part of the brain on the CT scan, accompanied by clinical worsening (e.g., drowsiness and increase of hemiparesis) or an increase of ≥4 points in the NIHSS score. The modified Rankin Scale (mRS) score at 90 days was assessed either by telephone or at a routine follow-up visit, with a poor outcome defined as a 90-day mRS score of 3–6 (20).

The thrombectomy strategies included stent retrieval (SR), contact aspiration (CA), a combination of SR with CA, or other methods. Other treatment modes included balloon angioplasty with or without stent placement.

The primary outcome was MBE. Based on previous studies (21–23), MBE was defined as a midline shift of ≥5 mm at the septum pellucidum or pineal gland with disappearance of the basal cisterns on follow-up head CT within 5 days of MT.

### Definition and evaluation of PCHDs

PCHDs were defined as higher densities increased by at least five Hounsfield units compared to that of the unaffected contralateral side on non-contrast computed tomography (NCCT) within 1 h after MT (11, 24).

**Figure 1:**
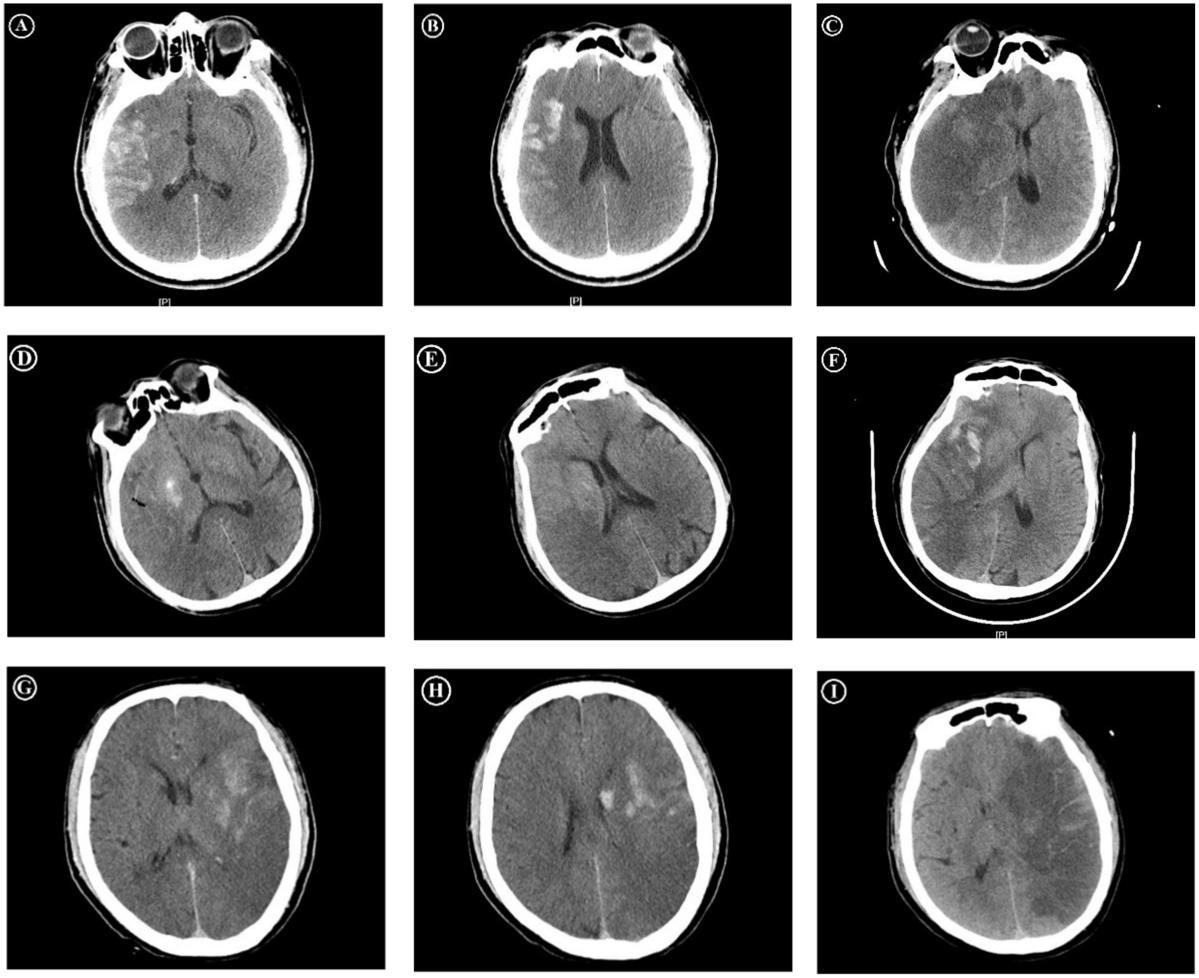
Immediate and follow-up CT images after MT. The first patient, who presented the cortex sign with PCHDS of 7 on CT immediately after MT **(A,B)**, developed MBE on follow-up CT **(C)**. The second patient, who presented the basal ganglia sign with PCHDS of 9 **(D,E)**, eventually developed MBE **(F)**. The third patient, who presented the combined sign with PCHDS of 7 **(G,H)**, eventually developed MBE **(I)**. MT, mechanical thrombectomy; MBE, malignant brain edema; PCHDS, postinterventional cerebral hyperdensities score.

The cortex–basal ganglia subtypes of PCHDs: PCHDs were further categorized into three subtypes according to their distribution characteristics. The cortex sign was characterized by higher densities confined exclusively to the cortical regions. The basal ganglia sign was marked by higher densities restricted solely to the subcortical regions. The combined sign was defined by the presence of higher densities in both the cortical and subcortical areas (Figure 1).

The scoring system of PCHDs: Postinterventional cerebral hyperdensities score (PCHDS) was used to assess the scope of PCHDs distribution. This score was based on ASPECTS, which divided the two levels of MCA territory into 10 regions. The total score was 10 points, with points deducted for each region affected by PCHDs (Figure 1).

### Statistical analysis

Continuous variables with normal distribution were summarized as mean ±standard deviation and analyzed using *t*-tests. Non-normally distributed continuous variables were summarized as median (interquartile range, [IQR]) and analyzed using the Mann–Whitney U-test. Categorical variables were expressed as numbers (percentages) and analyzed using the chi-squared test or Fisher’s exact test.

First, baseline information with and without MBE was described and compared among the patients included in this study. Second, the multivariate logistic regression analysis was performed to evaluate the predictive value of two PCHDs classification methods for MBE. The receiver operating characteristic (ROC) curve was used to analyze the optimal predictive value of the PCHDS for MBE. Finally, all patients were randomly divided into a training group and a test group at a ratio of 7:3. A nomogram was developed and internally validated in the training group, while external validation was conducted in the test group. Least absolute shrinkage and selection operator (LASSO) regression was applied to select significant predictors of MBE. Significant predictors were selected for the multivariate logistic regression analysis to construct the nomogram. If variance inflation factors (VIFs) were <5 and tolerances were >0.1, there was no significant collinearity between the variables. The performance of the nomogram was evaluated using the C-index, calibration chart, Hosmer–Lemeshow test, and decision curve analysis. Statistical analyses were conducted using IBM SPSS software version 26.0 and R 4.1.1 software, with a two-tailed *p*-value of < 0.05 considered statistically significant.

## Results

### Patient characteristics

This study included 516 AIS patients, among whom 310 (60.1%) presented with PCHDs, and 126 (24.4%) experienced MBE.

Supplementary Table S1 presents the baseline characteristics of the cortex–basal ganglia subtypes of PCHDs. We compared the cortex sign, basal ganglia sign, and combined sign individually with the PCHDs(−) group. The results showed statistically significant differences in MBE between any PCHDs(+) subgroup and the PCHDs(−) group (all *p* < 0.05).

Table 1 shows a comparison of demographic and clinical characteristics of patients with and without MBE. When it came to patients with MBE, they were more likely to have atrial fibrillation (*p* = 0.043), higher NIHSS scores (*p* < 0.001), lower ASPECTS (*p* < 0.001), higher preoperative diastolic blood pressure (*p* = 0.010), and higher blood glucose levels (*p* = 0.016). There were significant differences in white blood cell count (WBC), neutrophil to lymphocyte ratio (NLR), total cholesterol level, and glycosylated hemoglobin between the two groups (all *p* < 0.05). MBE patients had a higher proportion of ICA occlusion (*p* = 0.001) and more clot retrievals (*p* < 0.001). Compared to patients without MBE, the cortex sign and combined sign were more likely to be present in patients with MBE. Meanwhile, patients with MBE were more likely to have lower PCHDS (*p* < 0.001).

**Table 1:** Comparison of demographics and clinical characteristics between patients with and without MBE.

Characteristic	No-MBE (*n* = 390)	MBE (*n* = 126)	*p*-value
Age (year), median (IQR)	68.5 (58.0 ~ 76.0)	68.0 (59.8 ~ 75.0)	0.918
Sex			0.097
Male, *n* (%)	237 (60.8)	66 (52.4)	
Female, *n* (%)	153 (39.2)	60 (47.6)	
Hypertension, *n* (%)	260 (66.7)	86 (68.3)	0.742
Diabetes mellitus, *n* (%)	72 (18.5)	27 (21.4)	0.463
Atrial fibrillation, *n* (%)	164 (42.1)	66 (52.4)	0.043
Cardiovascular diseases, *n* (%)	69 (17.7)	20 (15.9)	0.639
Prior stroke, *n* (%)	57 (14.6)	24 (19.0)	0.235
Pre-stroke mRS score, median (IQR)	0.0 (0.0 ~ 0.0)	0.0 (0.0 ~ 0.0)	0.601
Smoking, *n* (%)	136 (34.9)	40 (31.7)	0.520
Alcohol consumption, *n* (%)	101 (25.9)	23 (18.3)	0.081
Baseline SBP (mm Hg), Median(IQR)	145.0 (131.0 ~ 161.0)	149.0 (135.0 ~ 166.5)	0.129
Baseline DBP (mm Hg), median (IQR)	83.0 (75.0 ~ 94.0)	89.0 (76.0 ~ 102.0)	0.010
Baseline blood glucose (mmol/L), median (IQR)	7.4 (6.5 ~ 8.9)	7.9 (6.7 ~ 9.9)	0.016
NIHSS score, Median (IQR)	16.0 (12.0 ~ 19.0)	19.0 (16.0 ~ 22.0)	<0.001
ASPECTS, Median (IQR)	7.0 (7.0 ~ 8.0)	7.0 (6.0 ~ 7.0)	<0.001
Intravenous thrombolysis, *n* (%)	131 (33.6)	39 (31.0)	0.584
WBC (10^9^/L), median (IQR)	9.2 (7.3 ~ 11.1)	11.6 (9.7 ~ 14.0)	<0.001
NLR, median (IQR)	7.3 (4.8 ~ 11.0)	12.8 (9.1 ~ 19.6)	<0.001
Monocyte (10^9^/L), median (IQR)	0.5 (0.3 ~ 0.6)	0.5 (0.3 ~ 0.6)	0.728
RBC (10^12^/L), median (IQR)	4.2 (3.8 ~ 4.6)	4.2 (3.8 ~ 4.7)	0.467
Hb (g/L), median (IQR)	129.0 (116.0 ~ 140.0)	128.0 (115.0 ~ 142.0)	0.724
PLT (10^9^/L), median (IQR)	193.0 (152.5 ~ 241.5)	188.0 (149.0 ~ 232.0)	0.458
TC (mmol/L), median (IQR)	4.1 (3.5 ~ 4.8)	4.4 (3.7 ~ 5.1)	0.014
HbA1c (%), median (IQR)	5.7 (5.4 ~ 6.2)	5.9 (5.5 ~ 6.7)	0.050
OPT (min), median (IQR)	290.0 (219.3 ~ 420.0)	300.0(230.0 ~ 350.0)	0.381
Stroke etiology			0.482
Large-artery atherosclerosis, *n* (%)	173(44.4)	48(38.1)	
Cardioembolism, *n* (%)	187(47.9)	73(57.9)	
Other etiology, *n* (%)	30(7.7)	5(4.0)	
Occlusion site			0.001
ICA, *n* (%)	91(23.3)	51 (40.5)	
MCA, *n* (%)	271 (69.5)	66 (52.4)	
Other^a^, *n* (%)	28 (7.2)	9 (7.1)	
Procedural modes			0.765
SR only, *n* (%)	135 (34.6)	41 (32.5)	
CA only, *n* (%)	74 (19.0)	17 (13.5)	
SR combined with CA, *n* (%)	91 (23.3)	46 (36.5)	
Other treatment modes^b^, *n* (%)	90 (23.1)	22 (17.5)	
Number of clot retrievals, median (IQR)	1.0 (1.0 ~ 2.0)	2.0 (1.0 ~ 3.0)	<0.001
Successful reperfusion, *n* (%)	361 (92.6)	110 (87.3)	0.069
Immediate CT imaging			<0.001
PCHDs(−), *n* (%)	191 (49.0)	15 (11.9)	
The cortex sign, *n* (%)	49 (12.6)	19 (15.1)	
The basal ganglia, *n* (%)	89 (22.8)	25 (19.8)	
The combined sign, *n* (%)	61 (15.6)	67 (53.2)	
PCHDS, median (IQR)	9.0 (8.0 ~ 10.00)	7.0 (5.0 ~ 9.0)	<0.001

mRS, modified Rankin Scale; SBP, systolic blood pressure; DBP, diastolic blood pressure; NIHSS, National Institute of Health Stroke Scale; ASPECTS, Alberta Stroke Program Early Computed Tomography Score; WBC, white blood cell; NLR, Neutrophil-to-Lymphocyte ratio; RBC, red blood cell; Hb, hemoglobin; PLT, platelet; TC, total cholesterol; HbA1c, hemoglobin A1c; OPT, onset to puncture time; ICA, internal carotid artery; MCA, middle cerebral artery; SR, stent retriever; CA, contact aspiration; MBE, malignant brain edema; PCHDs, postinterventional cerebral hyperdensities; PCHDS, postinterventional cerebral hyperdensities score; IQR, interquartile range. ^a^Other occlusion sites include carotid tandem lesions. ^b^Other treatment modes include balloon angioplasty with or without stent placement.

### Predictive value of PCHDs

A logistic regression analysis was performed with postoperative MBE as the outcome variable, and the results are shown in Table 2. An unadjusted univariate analysis showed that the cortex–basal ganglia subtypes of PCHDs and PCHDS were correlated with MBE (*p* < 0.001). This association persisted after adjusting for age and sex in model 1 (*p* < 0.001). Furthermore, there were still statistical differences after the multivariate logistic regression analysis using stepwise regression screening variables (*p* < 0.001). After adjusting for age, sex, atrial fibrillation, baseline NIHSS score, ASPECTS, white blood cell count, NLR, total cholesterol level, occlusion site, and number of clot retrievals, the risk of MBE was 4.081 times higher in patients with the basal ganglia sign [adjusted odds ratio (OR) = 4.081, 95% confidence interval (CI): 1.831–9.096], 6.290 times higher in patients with the cortex sign (adjusted OR = 6.290, 95% CI: 2.581–15.329), and 8.295 times higher in patients with the combined sign (adjusted OR = 8.295, 95% CI: 3.942–17.454) than that in PCHDs(−) patients. When PCHDS increased by 1 point, the risk of MBE decreased by 38% (adjusted OR = 0.620, 95% CI: 0.534–0.719).

**Table 2:** Relationship between postinterventional cerebral hyperdensities and malignant brain edema in different logistic regression models.

PCHDs	Unadjusted model		Model 1		Model 2	
	OR (95% CI)	*p*-value	OR (95% CI)	*p*-value	OR (95% CI)	*p*-value
PCHDs (−)	Reference		Reference		Reference	
The cortex sign	4.937 (2.341 ~ 10.413)	<0.001	4.918 (2.325 ~ 10.406)	<0.001	6.290 (2.581 ~ 15.329)	<0.001
The basal ganglia	3.577 (1.798 ~ 7.115)	<0.001	3.609 (1.811 ~ 7.193)	<0.001	4.081 (1.831 ~ 9.096)	<0.001
The combined sign	13.986 (7.451 ~ 26.252)	<0.001	14.348 (7.602 ~ 27.080)	<0.001	8.295 (3.942 ~ 17.454)	<0.001
PCHDS	0.557 (0.491 ~ 0.632)	<0.001	0.551 (0.485 ~ 0.626)	<0.001	0.620 (0.534 ~ 0.719)	<0.001

Model 1, Adjusted for age and gender; Model 2, Adjusted for age, gender, atrial fibrillation, NIHSS score, ASPECTS, WBC, NLR, TC, occlusion site, and number of clot retrieval.

### ROC curve analysis of the best predictive value

**Figure 2:**
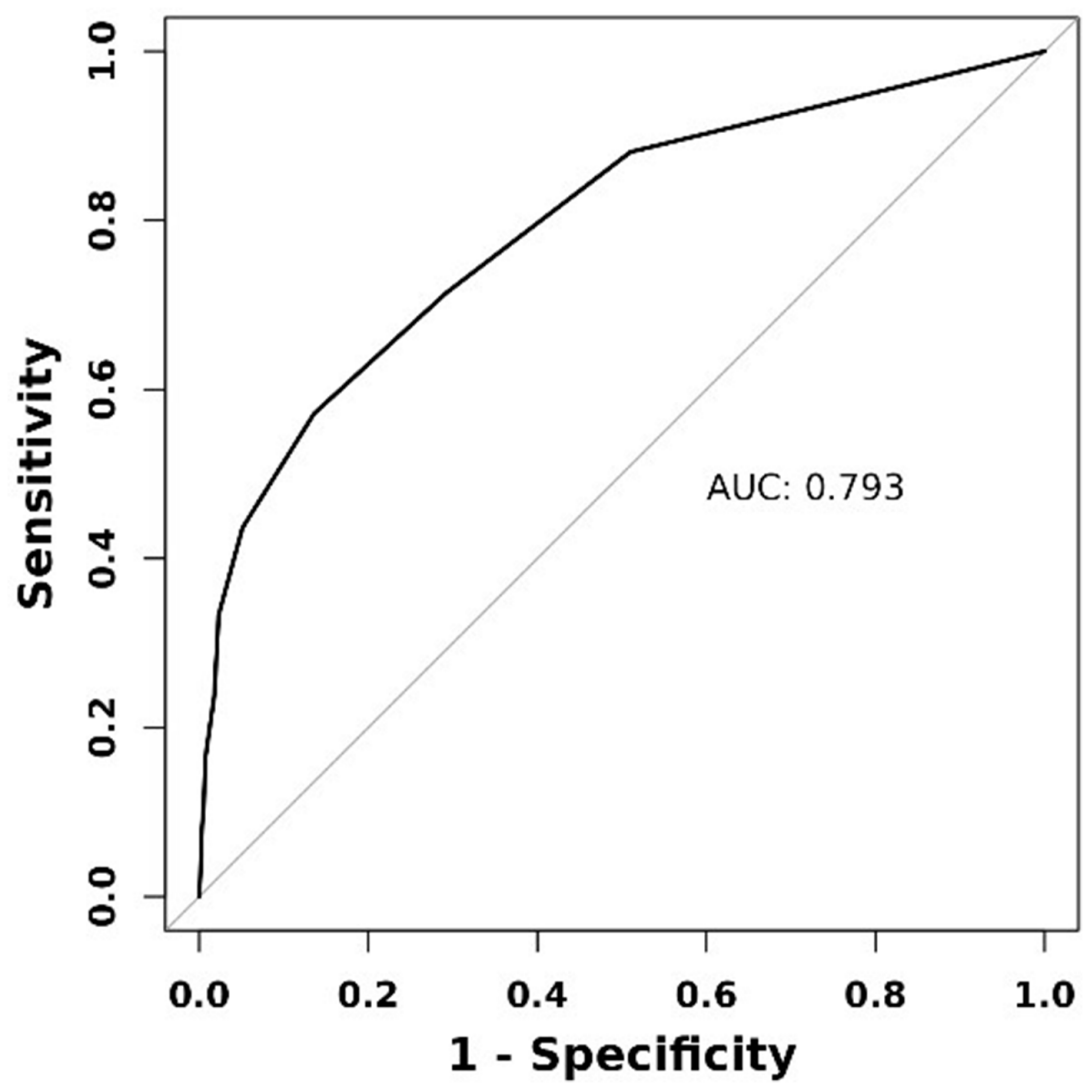
Receiver operating characteristic curve of PCHDS for predicting malignant brain edema.

The ROC curve showed that PCHDs had good discrimination for predicting MBE [area under the curve (AUC) = 0.793, 95% CI: 0.745–0.840; Figure 2]. The optimal cut-off value of PCHDS for predicting MBE was 7.5 (0–7 vs. 8–10), with a sensitivity of 86.4% and a specificity of 57.1%.

### Risk factors and nomograms for predicting MBE

Patients in our study were randomly divided into a training group (361 cases) and a test group (155 cases) in a 7:3 ratio. Supplementary Table S2 showed the baseline characteristics of the training and test groups, with no statistically significant difference in MBE rates between the two groups (23.8% vs. 25.8%, *p* = 0.631). Supplementary Table S3 compared demographics and clinical characteristics of patients with and without MBE in the training group.

λ_min_ was determined using LASSO regression and 10-fold cross validation, and 17 factors with non-zero coefficients were finally selected: age, atrial fibrillation, prior stroke, smoking history, intravenous thrombolysis, baseline blood glucose level, baseline NIHSS score, ASPECTS, white blood cell count, NLR, total cholesterol level, time from stroke onset to puncture (OPT), occlusion site, procedural modes, number of clot retrieval, successful reperfusion and PCHDS. The selected variables were included in the multivariate logistic regression analysis. After adjusting for prior stroke, smoking history, intravenous thrombolysis, baseline blood glucose level, ASPECTS, NLR, OPT, procedural modes, number of clot retrievals and successful reperfusion, the results showed that age (OR = 0.954, 95% CI: 0.928–0.981), atrial fibrillation (OR = 2.581, 95% CI: 1.204–5.536), baseline NIHSS score (OR = 1.069, 95% CI: 1.013–1.128), occlusion site (*p* < 0.01), white blood cell count (OR = 1.151, 95% CI: 1.031–1.285), total cholesterol level (OR = 1.430, 95% CI: 1.054–1.941), and PCHDS (OR = 0.566, 95% CI: 0.475–0.675) were independent risk factors for MBE (Table 3). Collinearity analysis suggested that there was no significant collinearity between the above variables (Supplementary Table S4).

**Table 3:** Significant predictors of MBE in acute ischemic stroke patients after MT from multivariate logistic regression analysis.

Variable	OR	95% CI	*p*-value
Age	0.954	0.928 ~ 0.981	0.001
Atrial fibrillation	2.581	1.204 ~ 5.536	0.015
NIHSS score	1.069	1.013 ~ 1.128	0.015
Occlusion site			0.002
ICA	Reference		
MCA	0.229	0.151 ~ 0.592	0.001
Other	0.864	0.240 ~ 3.108	0.823
WBC	1.151	1.031 ~ 1.285	0.012
TC	1.430	1.054 ~ 1.941	0.022
PCHDS	0.566	0.475 ~ 0.675	<0.001

NIHSS, National Institute of Health Stroke Scale; ICA, Internal carotid artery; MCA, Middle cerebral artery; WBC, White blood cell; TC, Total cholesterol; PCHDS, Postinterventional cerebral hyperdensities score; MBE, Malignant brain edema; MT, mechanical thrombectomy; OR, Odds ratio; CI, Confidence interval.

**Figure 3:**
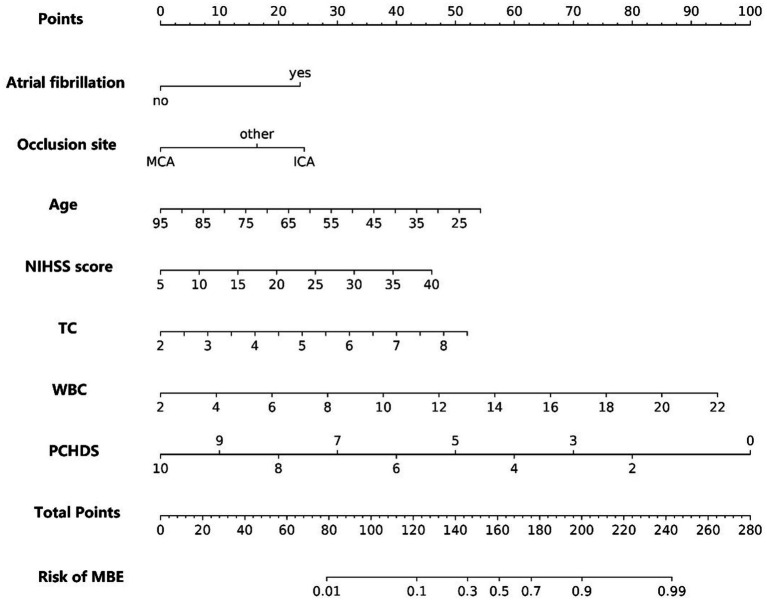
Nomogram for predicting the risk of malignant brain edema after mechanical thrombectomy in acute ischemic stroke patients.

According to the results of the multivariate logistic analysis, a nomogram predicting MBE in AIS patients after MT was drawn (Figure 3). The nomogram assigns scores to 7 independent risk factors. Each score is summed to calculate the total score, which is then converted into the individual risk ratio of MBE.

**Figure 4:**
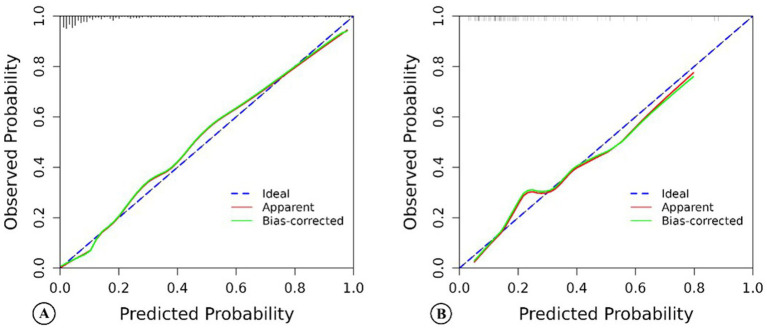
The calibration chart of the nomogram model predicting malignant brain edema of acute ischemic stroke patients in the training group **(A)** and the test group **(B)**.

**Figure 5:**
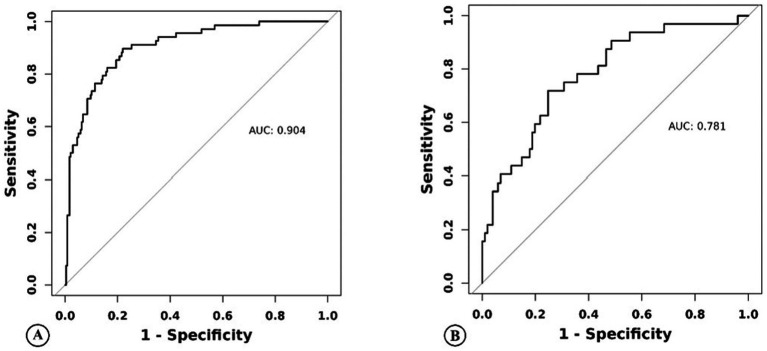
Nomogram model predicts the receiver operating characteristic curve of malignant brain edema of acute ischemic stroke patients in the training group **(A)** and the test group **(B)**.

**Figure 6:**
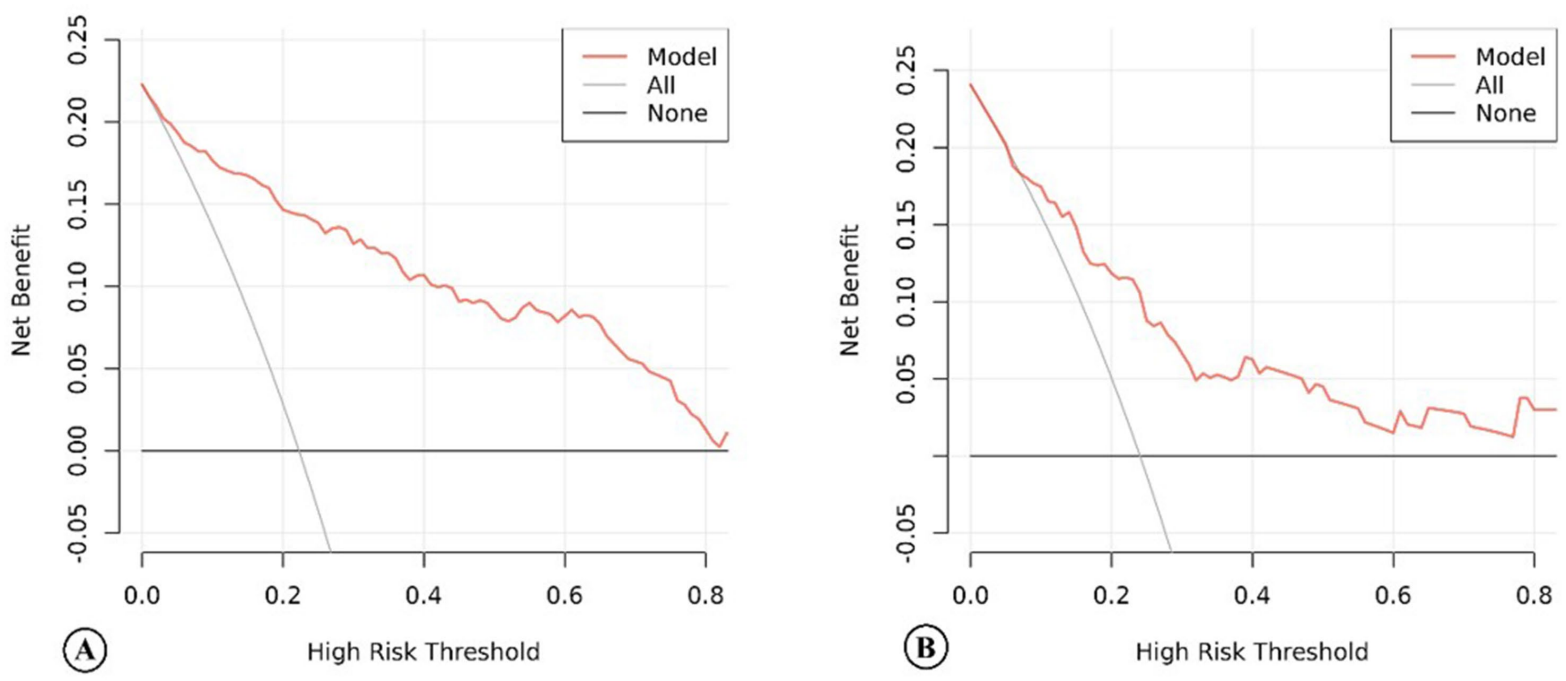
The decision curve analysis of the nomogram model of acute ischemic stroke patients in the training group **(A)** and the test group **(B)**.

For this model, the calibration plot (Figure 4) and the Hosmer–Lemeshow test (*χ*^2^ = 4.066, *p* = 0.851) suggested a good agreement between the observed and predicted probabilities of MBE. In addition, the C-index in the training group and the test group were 0.904 (95% CI: 0.864–0.944) and 0.781 (95% CI: 0.689–0.873), respectively. The ROC curve is shown in Figure 5, indicating that the nomogram model had good discrimination. Meanwhile, decision curve analysis was used to evaluate the net benefit under different medical intervention thresholds. Figure 6 suggested that this nomogram had good clinical utility.

## Discussion

This study demonstrated that PCHDs were strongly associated with MBE in AIS patients with anterior circulation large vessel occlusion treated with MT. PCHDS ≤7 predicted the occurrence of MBE with a sensitivity of 86.4% and a specificity of 57.1%. The nomogram incorporating age, atrial fibrillation, baseline NIHSS score, occlusion site, white blood cell count, total cholesterol level, and PCHDS could reliably and individually predict the risk of MBE after MT. However, this model needed to be validated by a multicenter study.

Other researchers found that the incidence of PCHDs ranged from 32.9 to 84.2% (11, 12). This wide variation might be attributed to differences in the definition of high-density lesions, the number of patients in each study, the quality of CT imaging equipment, and the time interval between recanalization and imaging. In our cohort, PCHDs occurred in 60.1% of patients, likely due to early post-MT imaging (≤1 h after MT), minimizing contrast clearance time.

Blood–brain barrier (BBB) disruption is the primary pathophysiological mechanism underlying PCHDs. In previous studies, the basal ganglia were found to be the most common site of PCHDs (25, 26). The most probable reason was that the basal ganglia were supplied by a variety of arteries, including the recurrent artery of Heubner, the lateral lenticulostriate artery, and the anterior choroidal artery, which were terminal arteries with poor collateral circulation. Due to the fragility of these vessels, the permeability of vessels in the basal ganglia was more easily damaged than in other parts of the brain (27). Therefore, another research (28) attempted to explore that the involvement of PCHDs in the cortical region beyond the basal ganglia may reflect more widespread BBB disruption. According to the above hypothesis, as the extent of BBB damage increased, the involvement of PCHDs progressively expanded from the basal ganglia to the cortical and combined regions, and the risk of MBE gradually increased. In our study, we observed that the cortex sign accounted for 21.9%, the basal ganglia sign accounted for 36.8%, and the combined sign accounted for 41.3% in the cortex–basal ganglia subtype of PCHDs. The higher proportion of the combined sign might be due to the relatively immature technology and equipment when thrombectomy was carried out in our hospital in 2017. Therefore, the incidence of the combined sign was relatively higher in the early stage due to the severe destruction of the BBB. In this study, we also obtained the same conclusion as the hypothesis: the risk of MBE was 4.081 times higher in patients with the basal ganglia sign, 6.290 times higher in patients with the cortex sign, and 8.295 times higher in patients with the combined sign than that in PCHDs(−) patients.

Previous studies had evaluated PCHDs by density, location, distribution, and volume (12, 28–30). Recent studies (31, 32) have applied ASPECTS to semi-quantitative assessment of the distribution scope of PCHDs in order to obtain a score. The majority of existing studies evaluate the correlation between this score and hemorrhagic transformation or final cerebral infarction volume, but few explore its relationship with MBE. In this study, the score was named PCHDS, with a total score of 10. The lower the PCHDS, the more the scope of PCHDs involved, the more severe the BBB disruption, and the higher the risk of MBE later. Our study also revealed a negative correlation between PCHDS and MBE risk: each 1-point increase in PCHDS corresponded to a 38% reduction in the risk of MBE. Meanwhile, ROC curve analysis indicated that PCHDS ≤ 7 could predict the occurrence of MBE in AIS patients after MT.

At present, known risk factors for MBE after MT include age, baseline NIHSS score, ASPECTS, successful reperfusion, collateral circulation status, and so on (21–23, 33, 34). More studies tend to establish multivariate risk prediction models based on demographic, clinical, and neuroimaging characteristics to predict MBE, such as the ACORNS score (22) and the EDEMA score (33). In this study, we attempted to establish a nomogram based on PCHDs for predicting MBE. The nomogram included age, atrial fibrillation, baseline NIHSS score, occlusion site, white blood cell count, total cholesterol level, and PCHDS. The correlation between age, baseline NIHSS score, and MBE was not surprising and confirmed in previous studies (7, 22). Age was negatively associated with the development of MBE, mainly because age-related brain atrophy might provide a buffer space for brain swelling. Conversely, a higher NIHSS score would indicate a poor collateral status and a larger infarct core, both of which were important risk factors for MBE. A history of atrial fibrillation was a common risk factor for cardioembolic stroke (35), accounting for 14–30% of all cerebral infarction (36, 37). Cardioembolic clots often embolize in areas such as atherosclerotic plaques, stenosis of the ICA, the intersection of the MCA and the anterior cerebral artery (ACA), and the upper and lower trunks of the MCA, which would lead to massive cerebral infarction and greatly increase the risk of MBE later. In addition, the sudden onset characteristic of cardioembolic stroke indicated that effective collateral circulation could not be established in time, resulting in poor collateral circulation and an increased risk of MBE. The relationship between occlusion site and MBE had rarely been explored. We found that ICA occlusion was an independent risk factor for the development of MBE. Similar results were also reported by Thomalla et al. (38) and Huang et al. (22) in a prospective multicenter study. This result could be explained that ICA occlusion might indicate the reduction of collateral blood flow supplied by the ACA or anterior communicating artery, thereby reducing collateral circulation. Additionally, clots blocked in the ICA are usually larger, which not only reduces successful recanalization rates but also prolongs operation time and increases onset-to-recanalization time. White blood cell count was identified as a predictor of MBE, as confirmed by numerous studies (39, 40). This was due to local inflammation in brain tissue resulting from BBB disruption, leading to peripheral immune cell infiltration and endothelial cell damage in AIS. We found a positive association between increased total cholesterol level and the incidence of MBE after MT. Hyperlipidemia was a prevalent risk factor for AIS. Approximately 45–60% of AIS patients exhibited elevated serum cholesterol levels in large-scale clinical trials and registry studies (41, 42). ElAli et al. (43) found that hyperlipidemia increased BBB permeability after focal cerebral ischemia, thereby aggravating cerebral edema.

Compared with the prior research, this study demonstrated advancement. The nomogram model converted risk factors into a graphical statistical tool with a continuous scoring system, allowing precise calculation of the risk probability of MBE for a certain patient. It plays an important role in the early identification of patients at risk for MBE and guides subsequent treatment strategies. In patients at high risk of MBE, it is essential to implement close clinical monitoring and a multidisciplinary treatment approach to reduce the adverse consequences of mass effect, including elevating the head of the bed to 30°, the appropriate use of sedatives, dehydration therapy to reduce intracranial pressure, and early decompressive craniotomy when necessary (44, 45). Second, this study incorporated PCHDs into the MBE prediction model for the first time and used the semi-quantitative evaluation method of PCHDs. Given that PCHDS more intuitively reflected the extent of BBB destruction after MT, our nomogram became a more convenient, accurate, and reliable prediction tool for patients with MBE.

This study has several limitations. First, the single-center retrospective design introduces potential selection bias. Second, this study did not compare this score with other established scores, such as the ACORNS score (22) and the EDEMA score (33). One important reason was the heterogeneity of patients enrolled and the different predictors used across different studies. Third, the contrast medium dosage during MT procedures was not analyzed in our study. A prior study (46) demonstrated a dose-dependent correlation between the contrast medium volume and PCHDs incidence. Fourth, dual-energy computed tomography (DECT) shows growing promise in stroke management. It not only distinguishes hemorrhage and contrast agent extravasation after endovascular treatment using virtual non-contrast (VNC) imaging and iodine overlay map (IOM) (47), but also detects brain edema and predicts future cerebral infarction volume through edema maps (48). Therefore, DECT has a unique advantage in predicting delayed MBE. However, DECT availability remains restricted in most clinical settings. Furthermore, the edema map lacks standardized parameters and remains investigational. Therefore, DECT-related data were not included in this study. Finally, it is important to note that the wide confidence intervals for key outcome estimates indicate substantial uncertainty, and these results should be applied with caution in clinical practice.

## Conclusion

PCHDs are common imaging findings on head CT scans in patients with acute anterior circulation large vessel occlusion stroke after MT. PCHDs are closely associated with the development of MBE. The nomogram based on PCHDs provides a reliable and individualized prediction of MBE risk in AIS patients after MT. However, multicenter validation is required before clinical implementation. In patients with high MBE risk, intensive monitoring and timely interventions are critical to mitigate mortality and improve long-term functional outcomes.

## Data Availability

The raw data supporting the conclusions of this article will be made available by the authors, without undue reservation.

## Glossary

ACA: Anterior cerebral artery
AIS: Acute ischemic stroke
ASPECTS: Alberta Stroke Program Early CT Score
BBB: Blood–brain barrier
CA: Contact aspiration
CI: Confidence interval
DSA: Digital subtraction angiography
DECT: Dual-energy computed tomography
ECASS-II: European Cooperative Acute Stroke Study II
ICA: Internal carotid artery
IOM: Iodine overlay map
IQR: Interquartile range
LASSO: Least absolute shrinkage and selection operator
MBE: Malignant brain edema
MCA: Middle cerebral artery
mRS: Modified Rankin Scale
MT: Mechanical thrombectomy
mTICI: Modified thrombolysis in cerebral infarction
NCCT: Non-contrast computed tomography
NIHSS: National Institute of Health Stroke Scale
NLR: Neutrophil-to-lymphocyte ratio
OPT: Time from stroke onset to puncture
OR: Odds ratio
PCHDs: Postinterventional cerebral hyperdensities
PCHDS: Postinterventional cerebral hyperdensities score
ROC: Receiver operating characteristic
sICH: Symptomatic intracranial hemorrhage
SR: Stent retrieval
TOAST: Trial of Org 10,172 in Acute Stroke Treatment
VIF: Variance inflation
VNC: Virtual non-contrast
