# Supplementary material for: Relationship between postinterventional cerebral hyperdensities and malignant brain edema in patients with acute ischemic stroke after mechanical thrombectomy
Source: Front Neurol. 2025 Oct 3;16:1693606. doi: 10.3389/fneur.2025.1693606 (PMC12533281; doi:10.3389/fneur.2025.1693606)
Supplement: Supplementary file 1 [file Table_1.docx]

**Supplementary table 1** The baseline characteristics of the cortex-basal ganglia subtypes of PCHDs

| Characteristic | PCHDs(-)  (n=206) | PCHDs(+) | | | | | |
| --- | --- | --- | --- | --- | --- | --- | --- |
|  |  | The cortex sign  (n=68) | | The basal ganglia sign  (n=114) | | The combined sign  (n=128) | |
|  |  | Data | *P* Value | Data | *P* Value | Data | *P* Value |
| Age(yr), Median(IQR) | 68.0(57.0~75.3) | 69.5(57.3~79.0) | 0.266 | 67.0(58.0~75.0) | 0.931 | 70.0(60.0~76.0) | 0.152 |
| Gender |  |  | 0.233 |  | 0.911 |  | 0.375 |
| Male, n(%) | 126(61.2) | 36(52.9) |  | 69(60.5) |  | 72(56.3) |  |
| Female, n(%) | 80(38.8) | 32(47.1) |  | 45(39.5) |  | 56(43.8) |  |
| Hypertension, n(%) | 138(67.0) | 49(72.1) | 0.437 | 67(58.8) | 0.143 | 92(71.9) | 0.349 |
| Diabetes mellitus, n(%) | 40(19.4) | 10(14.7) | 0.384 | 25(21.9) | 0.593 | 24(18.8) | 0.880 |
| Atrial fibrillation, n(%) | 78(37.9) | 39(57.4) | 0.005 | 51(44.7) | 0.231 | 62(48.4) | 0.057 |
| Cardiovascular diseases, n (%) | 36(17.5) | 14(20.6) | 0.565 | 19(16.7) | 0.854 | 20(15.6) | 0.660 |
| Prior stroke, n(%) | 28(13.6) | 10(14.7) | 0.818 | 17(14.9) | 0.745 | 26(20.3) | 0.105 |
| Smoking, n(%) | 75(36.4) | 21(30.9) | 0.408 | 38(33.3) | 0.582 | 42(32.8) | 0.504 |
| Alcohol consumption, n(%) | 55(26.7) | 13(19.1) | 0.210 | 28(24.6) | 0.677 | 28(21.9) | 0.322 |
| Baseline SBP(mmHg), Median(IQR) | 145.0(131.0~162.0) | 147.0(133.5~160.0) | 0.598 | 142.5(128.0~165.3) | 0.892 | 149.0(135.0~162.0) | 0.265 |
| Baseline DBP(mmHg), Median(IQR) | 82.0(74.0~74.0) | 85.5(78.0~98.0) | 0.081 | 84.5(75.0~97.3) | 0.406 | 86.0(75.5~100.0) | 0.066 |
| Baseline blood glucose(mmol/L), Median (IQR) | 6.3(5.3~8.2) | 6.9(5.9~8.7) | 0.585 | 6.0(5.4~7.8) | 0.914 | 7.7(6.5~9.9) | 0.045 |
| NIHSS score, Median (IQR) | 15.5(12.0~19.0) | 17.0(14.0~19.0) | 0.028 | 15.0(13.0~20.0) | 0.335 | 18.0(15.0~21.0) | <0.001 |
| ASPECTS, Median (IQR) | 7.0(7.0~8.0) | 7.0(7.0~8.0) | 0.042 | 7.0(7.0~8.0) | 0.005 | 7.0(6.0~7.0) | <0.001 |
| Intravenous thrombolysis, n(%) | 65(31.6) | 21(30.9) | 0.918 | 38(33.3) | 0.745 | 46(35.9) | 0.409 |
| WBC(10^9^/L), Median (IQR) | 9.4(7.5~11.6) | 9.5(7.2~11.6) | 0.818 | 9.1(7.5~11.4) | 0.896 | 11.2(9.0~13.5) | <0.001 |
| NLR, Median(IQR) | 7.4(4.5~10.7) | 10.3(6.5~14.6) | <0.001 | 7.9(5.2~10.9) | 0.475 | 11.5(6.6~19.5) | <0.001 |
| Monocyte(10^9^/L), Median (IQR) | 0.5(0.4~0.6) | 0.4(0.3~0.6) | 0.134 | 0.4(0.3~0.6) | 0.067 | 0.5(0.4~0.6) | 0.875 |
| RBC(10^12^/L), Median (IQR) | 4.3(3.9~4.6) | 4.1(3.9~4.5) | 0.223 | 4.2(3.7~4.6) | 0.236 | 4.3(3.8~4.7) | 0.954 |
| Hb(g/L), Median (IQR) | 130.0(117.8~140.3) | 128.0(115.0~138.8) | 0.430 | 126.0(114.0~140.0) | 0.555 | 128.0(117.8~143.0) | 0.784 |
| PLT(10^9^/L), Median (IQR) | 203.0(169.0~244.3) | 185.0(135.5~234.8) | 0.027 | 191.5(151.0~231.8) | 0.040 | 174.0(136.8~227.0) | <0.001 |
| TC(mmol/L), Median(IQR) | 4.1(3.5~4.9) | 3.9(3.2~4.7) | 0.165 | 4.3(3.7~4.8) | 0.341 | 4.3(3.9~5.2) | 0.023 |
| HbA1c(%), Median (IQR) | 5.7(5.4~6.4) | 5.6(5.3~6.0) | 0.116 | 5.7(5.4~6.4) | 0.851 | 5.9(5.6~6.6) | 0.093 |
| OPT(min), Median(IQR) | 294.0(216.0~413.5) | 310.0(238.8~430.0) | 0.551 | 290.0(212.5~391.0) | 0.534 | 282.0(223.5~346.5) | 0.382 |
| Stroke etiology |  |  | 0.276 |  | 0.827 |  | 0.993 |
| Large-artery atherosclerosis, n (%) | 93(45.1) | 24(35.3) |  | 51(44.7) |  | 53(41.4) |  |
| Cardioembolism, n (%) | 94(45.6) | 39(57.4) |  | 56(49.1) |  | 71(55.5) |  |
| Other etiology, n (%) | 19(9.2) | 5(7.4) |  | 7(6.1) |  | 4(3.1) |  |
| Occlusion site |  |  | 0.042 |  | 0.163 |  | 0.935 |
| ICA, n(%) | 65(31.6) | 10(14.7) |  | 26(22.8) |  | 41(32.0) |  |
| MCA, n(%) | 126(61.2) | 55(80.9) |  | 80(70.2) |  | 76(59.4) |  |
| Other, n(%) | 15(7.3) | 3(4.4) |  | 8(7.0) |  | 11(8.6) |  |
| Procedural modes |  |  | 0.650 |  | 0.878 |  | 0.942 |
| SR only, n (%) | 70(34.0) | 26(38.2) |  | 37(32.5) |  | 43(33.6) |  |
| CA only, n (%) | 41(19.9) | 11(16.2) |  | 21(18.4) |  | 18(14.1) |  |
| SR combined with CA, n (%) | 45(21.8) | 16(23.5) |  | 31(27.2) |  | 45(35.2) |  |
| Other treatment modes†, n (%) | 50(24.3) | 15(22.1) |  | 25(21.9) |  | 22(17.2) |  |
| Number of clot retrieval, Median (IQR) | 1.0(1.0~2.0) | 2.0(1.0~3.0) | 0.007 | 2.0(1.0~3.0) | 0.031 | 2.0(1.0~3.0) | <0.001 |
| Successful reperfusion, n(%) | 179(86.9) | 60(88.2) | 0.774 | 108(94.7) | 0.027 | 124(96.9) | 0.002 |
| PCHDS, Median(IQR) | 10.0(10.0~10.0) | 8.0(8.0~9.0) | <0.001 | 9.0(8.0~9.0) | <0.001 | 6.0(4.0~7.0) | <0.001 |
| sICH, n(%) | 1(0.5) | 13(19.1) | <0.001 | 20(17.5) | <0.001 | 59(46.1) | <0.001 |
| MBE, n(%) | 15(7.3) | 19(27.9) | <0.001 | 25(21.9) | <0.001 | 67(52.3) | <0.001 |
| Poor outcome, n(%) | 95(46.1) | 44(64.7) | 0.008 | 68(59.6) | 0.021 | 111(86.7) | <0.001 |

Note: SBP: systolic blood pressure; DBP: diastolic blood pressure; NIHSS: National Institute of Health Stroke Scale; ASPECTS: Alberta Stroke Program Early Computed Tomography Score; WBC: white blood cell; NLR: Neutrophil-to-Lymphocyte ratio; RBC: red blood cell; Hb: hemoglobin; PLT: platelet; TC: total cholesterol; HbA1c: hemoglobin A1c; OPT: onset to puncture time; ICA: internal carotid artery; MCA: middle cerebral artery; SR: stent retriever; CA: contact aspiration; sICH: symptomatic intracranial hemorrhage; MBE: malignant brain edema; PCHDs: postinterventional cerebral hyperdensities; PCHDS: postinterventional cerebral hyperdensities score; IQR: interquartile range.

†Other treatment modes include balloon angioplasty with or without stent placement.

**Supplementary table 2** Demographic and clinical characteristics of acute ischemic stoke patients in the training group and the test group

| Characteristic | The training group  (n=361) | The test group  (n=155) | *P* value |
| --- | --- | --- | --- |
| Age(yr), Median(IQR) | 68.0(58.0~76.0) | 69.0(59.0~77.0) | 0.494 |
| Gender |  |  | 0.331 |
| Male, n(%) | 207(57.3) | 96(61.9) |  |
| Female, n(%) | 154(42.7) | 59(38.1) |  |
| Hypertension, n(%) | 234(64.8) | 112(72.3) | 0.099 |
| Diabetes mellitus, n(%) | 68(18.8) | 31(20.0) | 0.758 |
| Atrial fibrillation, n(%) | 160(44.3) | 70(45.2) | 0.860 |
| Cardiovascular diseases, n (%) | 63(17.5) | 26(16.8) | 0.852 |
| Prior stroke, n(%) | 55(15.2) | 26(16.8) | 0.660 |
| Smoking, n(%) | 127(35.2) | 49(31.6) | 0.433 |
| Alcohol consumption, n(%) | 88(24.4) | 36(23.2) | 0.779 |
| Baseline SBP(mmHg), Median(IQR) | 145.0(130.0~160.0) | 150.0(135.0~165.0) | 0.024 |
| Baseline DBP(mmHg), Median(IQR) | 83.0(75.0~95.0) | 88.0(77.0~101.0) | 0.023 |
| Baseline blood glucose(mmol/L), Median (IQR) | 7.5(6.5~8.9) | 7.7(6.6~9.2) | 0.198 |
| NIHSS score, Median (IQR) | 17.0(13.0~20.0) | 16.0(13.0~20.0) | 0.856 |
| ASPECTS, Median (IQR) | 7.0(7.0~8.0) | 7.0(7.0~8.0) | 0.638 |
| Intravenous thrombolysis, n(%) | 114(31.6) | 56(36.1) | 0.313 |
| WBC(10^9^/L), Median (IQR) | 9.7(7.7~12.2) | 9.8(7.9~12.1) | 0.827 |
| NLR, Median(IQR) | 8.0(5.0~13.0) | 9.0(5.0~13.0) | 0.541 |
| Monocyte(10^9^/L), Median (IQR) | 0.5(0.3~0.6) | 0.5(0.3~0.6) | 0.310 |
| RBC(10^12^/L), Median (IQR) | 4.2(3.8~4.6) | 4.2(3.9~4.6) | 0.388 |
| Hb(g/L), Median (IQR) | 128.0(115.0~141.0) | 131.0(119.0~140.0) | 0.230 |
| PLT(10^9^/L), Median (IQR) | 189.0(149.0~240.0) | 197.0(161.0~233.0) | 0.362 |
| TC(mmol/L), Median(IQR) | 4.2(3.6~4.9) | 4.0(3.5~4.8) | 0.304 |
| HbA1c(%), Median (IQR) | 5.7(5.4~6.3) | 5.9(5.5~6.6) | 0.083 |
| OPT(min), Median(IQR) | 300.0(220.0~385.0) | 276.0(229.0~397.0) | 0.730 |
| Stroke etiology |  |  | 0.837 |
| Large-artery atherosclerosis, n (%) | 152(42.1) | 69(44.5) |  |
| Cardioembolism, n (%) | 185(51.2) | 75(48.4) |  |
| Other etiology, n (%) | 24(6.6) | 11(7.1) |  |
| Occlusion site |  |  | 0.918 |
| ICA, n(%) | 99(27.4) | 43(27.7) |  |
| MCA, n(%) | 235(65.1) | 102(65.8) |  |
| Other, n(%) | 27(7.5) | 10(6.5) |  |
| Procedural modes |  |  | 0.497 |
| SR only, n (%) | 119(33.0) | 57(36.8) |  |
| CA only, n (%) | 60(16.6) | 31(20.0) |  |
| SR combined with CA, n (%) | 101(28.0) | 36(23.2) |  |
| Other treatment modes†, n (%) | 81(22.4) | 31(20.0) |  |
| Number of clot retrieval, Median (IQR) | 2.0(1.0~3.0) | 1.0(1.0~2.0) | 0.028 |
| Successful reperfusion, n(%) | 326(90.3) | 145(93.5) | 0.231 |
| PCHDS, Median(IQR) | 9.0(8.0~10.0) | 9.0(7.0~10.0) | 0.517 |
| MBE, n(%) | 86(23.8) | 40(25.8) | 0.631 |

Note: SBP: systolic blood pressure; DBP: diastolic blood pressure; NIHSS: National Institute of Health Stroke Scale; ASPECTS: Alberta Stroke Program Early Computed Tomography Score; WBC: white blood cell; NLR: Neutrophil-to-Lymphocyte ratio; RBC: red blood cell; Hb: hemoglobin; PLT: platelet; TC: total cholesterol; HbA1c: hemoglobin A1c; OPT: onset to puncture time; ICA: internal carotid artery; MCA: middle cerebral artery; SR: stent retriever; CA: contact aspiration; MBE: malignant brain edema; PCHDS: postinterventional cerebral hyperdensities score; IQR: interquartile range.

†Other treatment modes include balloon angioplasty with or without stent placement.

**Supplementary table 3** Compatision of demographic and clinical characteristics of patients with and without MBE in the training group of acute ischemic stroke patients

| Characteristic | No-MBE  (n=275) | MBE  (n=86) | *P* value |
| --- | --- | --- | --- |
| Age(yr), Median(IQR) | 68.0(58.0~76.0) | 68.0(60.0~74.0) | 0.770 |
| Gender |  |  | 0.184 |
| Male, n(%) | 163(59.3) | 44(51.2) |  |
| Female, n(%) | 112(40.7) | 42(48.8) |  |
| Hypertension, n(%) | 174(63.3) | 60(69.8) | 0.271 |
| Diabetes mellitus, n(%) | 51(18.5) | 17(19.8) | 0.800 |
| Atrial fibrillation, n(%) | 113(41.1) | 47(54.7) | 0.027 |
| Cardiovascular diseases, n (%) | 48(17.5) | 15(17.4) | 0.998 |
| Prior stroke, n(%) | 41(14.9) | 14(16.3) | 0.758 |
| Smoking, n(%) | 96(34.9) | 31(36.0) | 0.847 |
| Alcohol consumption, n(%) | 73(26.5) | 15(17.4) | 0.086 |
| Baseline SBP(mmHg), Median(IQR) | 145.0(130.0~159.0) | 146.0(126.0~165.0) | 0.549 |
| Baseline DBP(mmHg), Median(IQR) | 82.0(75.0~92.0) | 88.0(75.0~101.0) | 0.042 |
| Baseline blood glucose(mmol/L), Median (IQR) | 7.4(6.5~8.6) | 7.9(6.7~9.9) | 0.018 |
| NIHSS score, Median (IQR) | 16.0(12.0~19.0) | 18.0(16.0~22.0) | <0.001 |
| ASPECTS, Median (IQR) | 7.0(7.0~8.0) | 7.0(6.0~7.0) | <0.001 |
| Intravenous thrombolysis, n(%) | 90(32.7) | 24(27.9) | 0.401 |
| WBC(10^9^/L), Median (IQR) | 9.1(7.3~11.1) | 12.2(10.1~14.0) | <0.001 |
| NLR, Median(IQR) | 7.0(5.0~11.0) | 13.0(9.0~21.0) | <0.001 |
| Monocyte(10^9^/L), Median (IQR) | 0.5(0.3~0.6) | 0.5(0.4~0.7) | 0.328 |
| RBC(10^12^/L), Median (IQR) | 4.2(3.8~4.6) | 4.2(3.8~4.7) | 0.494 |
| Hb(g/L), Median (IQR) | 129.0(115.0~140.0) | 128.0(114.0~143.0) | 0.833 |
| PLT(10^9^/L), Median (IQR) | 191.0(150.0~242.0) | 187.0(143.0~231.0) | 0.634 |
| TC(mmol/L), Median(IQR) | 4.1(3.5~4.7) | 4.6(3.9~5.2) | 0.002 |
| HbA1c(%), Median (IQR) | 5.7(5.4~6.2) | 5.8(5.5~6.5) | 0.090 |
| OPT(min), Median(IQR) | 300.0(211.0~416.0) | 299.0(224.0~345.0) | 0.208 |
| Stroke etiology |  |  | 0.139 |
| Large-artery atherosclerosis, n (%) | 122(44.4) | 30(34.9) |  |
| Cardioembolism, n (%) | 133(48.4) | 52(60.5) |  |
| Other etiology, n (%) | 20(11.4) | 4(4.7) |  |
| Occlusion site |  |  | 0.003 |
| ICA, n(%) | 63(22.9) | 36(41.9) |  |
| MCA, n(%) | 190(69.1) | 45(52.3) |  |
| Other, n(%) | 22(8.0) | 5(5.8) |  |
| Procedural modes |  |  | 0.041 |
| SR only, n (%) | 88(32.0) | 31(36.0) |  |
| CA only, n (%) | 51(18.5) | 9(10.5) |  |
| SR combined with CA, n (%) | 69(25.1) | 32(37.2) |  |
| Other treatment modes†, n (%) | 67(24.4) | 14(16.3) |  |
| Number of clot retrieval, Median (IQR) | 1.0(1.0~2.0) | 2.0(1.0~3.0) | <0.001 |
| Successful reperfusion, n(%) | 253(92.0) | 73(84.9) | 0.052 |
| PCHDS, Median(IQR) | 9.0(8.0~10.0) | 7.0(5.0~9.0) | <0.001 |

Note: SBP: systolic blood pressure; DBP: diastolic blood pressure; NIHSS: National Institute of Health Stroke Scale; ASPECTS: Alberta Stroke Program Early Computed Tomography Score; WBC: white blood cell; NLR: Neutrophil-to-Lymphocyte ratio; RBC: red blood cell; Hb: hemoglobin; PLT: platelet; TC: total cholesterol; HbA1c: hemoglobin A1c; OPT: onset to puncture time; ICA: internal carotid artery; MCA: middle cerebral artery; SR: stent retriever; CA: contact aspiration; MBE: malignant brain edema; PCHDS: postinterventional cerebral hyperdensities score; IQR: interquartile range.

†Other treatment modes include balloon angioplasty with or without stent placement.

**Supplementary table 4** The statistical collinearity analysis of the variables included in the nomogram

| Variable | Tolerance | VIF |
| --- | --- | --- |
| Age | 0.718 | 1.393 |
| Atrial fibrillation | 0.712 | 1.404 |
| NIHSS score | 0.961 | 1.041 |
| Occlusion site | 0.856 | 1.168 |
| WBC | 0.899 | 1.113 |
| TC | 0.929 | 1.077 |
| PCHDS | 0.920 | 1.086 |

Note: NIHSS: National Institute of Health Stroke Scale; WBC: white blood cell; TC: total cholesterol; PCHDS: postinterventional cerebral hyperdensities score; VIF: variance inflation factor.
